# Application of the ultrasound-guided double-screen contrast method in the standardized teaching and training of resident doctors in femoral vein puncture

**DOI:** 10.3389/fcvm.2022.1026619

**Published:** 2023-01-19

**Authors:** Yunshui Zhang, Jin Li, Yaying Sun, Tao Liu, Wenmin Guo, Weiwei Ban, Yujie Ma

**Affiliations:** ^1^Department of Critical Care Medicine, Air Force Characteristic Medical Center, Air Force Medical University, Beijing, China; ^2^Department of Otorhinolaryngology, Yutian Hospital, Tangshan, Hebei, China

**Keywords:** ultrasound-guided, puncture of femoral vein, double-screen contrast, application, teaching and training of resident doctors

## Abstract

**Objective:**

The aim of this study was to investigate the advantages of the double-screen contrast method compared with the short-axis ultrasound display method for teaching ultrasound-guided femoral vein puncture during the standardized training of resident doctors.

**Methods:**

Sixty resident doctors undergoing standardized training were randomly divided into a test group (short–long axis switching double-screen contrast, *n* = 30) and a control group (*n* = 30). These two groups of physicians underwent teaching and training of ultrasound-guided femoral vein puncture, and the success rate of the first puncture attempt, the total catheterization time, and any accidental femoral artery punctures were recorded and compared between the two groups.

**Results:**

The success rate of the first puncture attempt in the test group was significantly higher than that in the control group (*P* < 0.05). In the control group, two doctors accidentally punctured the artery, while in the test group, no arterial punctures occurred. The puncture time for the test group was longer than for the control group (*P* < 0.05).

**Conclusion:**

In the standardized training of ultrasound-guided femoral vein puncture for resident doctors, the double-screen contrast method has significant advantages. It enables resident doctors to quickly understand and apply the technique, so it is worth making this the method of choice.

## 1. Introduction

The Air Force Characteristic Medical Center is one of the first groups of standardized training bases for resident doctors recognized by the National Health and Family Planning Commission. According to the requirements for standardized training of resident doctors, these doctors need to master the technique of deep vein puncture and catheterization. With the increased use of bedside ultrasound technology, vascular puncture has gradually become a visualized operation in clinic. Some studies have revealed that ultrasound-guided deep vein puncture could significantly reduce complications and improve the puncture success rate (1, 2). In clinical teaching, application of the ultrasound-guided technique has gradually become more popular (3). However, there are few studies concerning teaching the ultrasound-guided deep vein puncture technique to resident doctors. The short–long axis switching double-screen method was found to work well in clinical teaching. In the process of applying this method, the “contrast” was used as a scan of the target vessel in different sections to guide central vein puncture. Unlike the enhanced scan in traditional examination, this method does not require the use of additional contrast media to enhance angiography. The results of this research are summarized below.

## 2. Information and methods

### 2.1. General information

According to the standard random number table grouping method, 60 resident doctors who were receiving standardized training in the Air Force Characteristic Medical Center were enrolled and randomly divided into a test group (*n* = 30) and a control group (*n* = 30). The number of samples in each group was calculated by PASS, showing 25–30 residents in each group is theoretically enough to tell the difference. The ultrasound machine used was an Edge ultrasound diagnostic system (Sonosite, USA), with 5–10 MHz high-frequency ultrasonic probes. In order to reduce clinical bias, all patients in this teaching and research were subjected to ultrasound-guided femoral venipuncture under analgesia and sedation. Moreover, the residents were allocated to perform the puncture in a single patient per resident.

### 2.2. Methods

First, each resident doctor was given a questionnaire. The questions covered whether the respondent had experience of ultrasound-guided femoral vein puncture, the adjacent positioning of the femoral vein and artery, and the complications of femoral vein puncture to determine whether there was a difference in the level of knowledge between the two groups before training. Before the experiment, the same teacher reviewed the anatomy of the lower limbs and conducted simulated operation training with both groups of resident doctors. All patients were placed in a supine position with the head elevated by 30°. Routine disinfection and local anesthesia were applied. In this puncture teaching, residents stood on the right side of patients to perform right femoral vein puncture. The puncture operation was stopped after the third unsuccessful attempt or if the artery was accidentally punctured, and this was regarded as a failed operation. For the former, senior physicians of our department continue to perform ipsilateral puncture to complete the teaching demonstration. For the latter, the artery that has been mistakenly penetrated needs a long time of compression and hemostasis. At this time, senior physicians in our department selected the left femoral vein for puncture, and completed the teaching demonstration.

In the control group, the out-of-plane ultrasound-guided method was used to teach femoral vein puncture. The ultrasound probe was placed perpendicular to the long axis of the lower limb and pointed under the inguinal ligament to find the target blood vessel. Attention was paid to the evaluation of the thickness and course of the femoral vein, the elasticity of the vascular wall, the rate of blood flow, the presence or absence of thrombus, and the relationship with the femoral artery and surrounding tissues. Identification of the artery and vein was emphasized. The wall of the femoral vein is thin, the lumen is mostly elliptical, pulsation is absent, and it can be flattened by pressing the probe against the skin. The wall of the femoral artery is thick, the lumen is round, pulsation can be seen, and compression cannot flatten it. The check focus was moved to the center of the probe along the femoral vein, and 0.5 cm from the center of the probe was the puncture point. The puncture needle was inserted at a 45° angle. When the puncture needle entered the femoral vein, dark red blood flowed out after suction. The end of the puncture needle was inserted into the tube connected to the infusion device. (The saline injection had been prepared in advance. A needle was initially connected to the infusion device, and then the needle was replaced with a tube, and the gas was expelled.) When the infusion was observed to have occurred successfully, a guide wire was inserted, and the puncture needle was withdrawn. Ultrasound was used to check that the guide wire was in the femoral vein. Then the skin was stretched, and a deep venous catheter was inserted. Finally, the catheter was locked with heparin water and taped into position.

**FIGURE 1:**
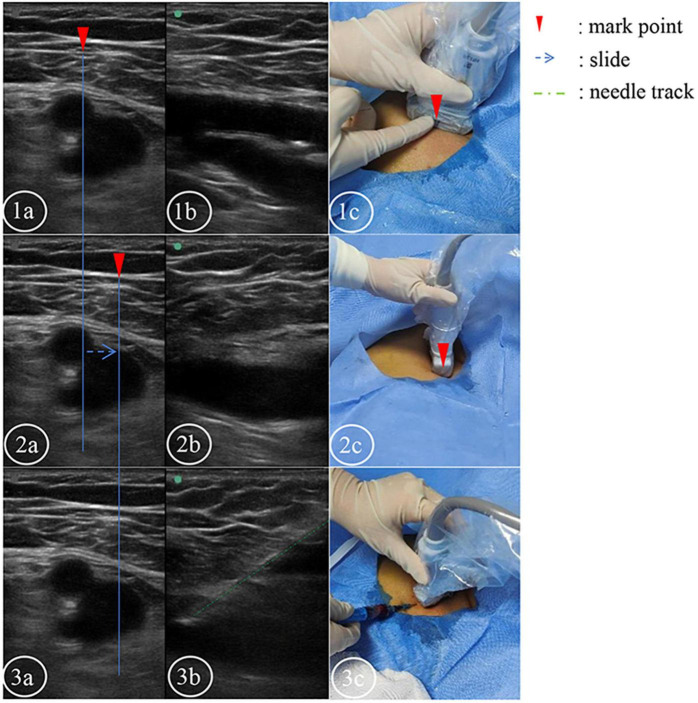
The short and long axis double screens were used to control the puncture plane. **(1–3a)** Short-axis plane. **(1–3b)** Long axis plane. The corresponding body surface real-time ultrasound probe in picture **(1c)**. The corresponding body surface real-time ultrasound probe in picture **(2c)**. **(3b)** Plane of venipuncture. The green line indicates the needle trajectory. **(3c)** Real-time ultrasound probe on the body surface at the time of puncture.

In the test group, the short–long axis switching double-screen contrast method was used to teach femoral vein puncture. The ultrasound probe was placed perpendicular to the lower limbs, and a suitable section was found under the inguinal ligament to display the femoral artery and vein in the center of the screen simultaneously. On the short-axis plane, the artery and vein can easily be distinguished due to their relative positions, the presence of a pulse in the artery, and the differential effects of pressure changes. The double-screen mode was adopted, and the left screen displayed the standard short-axis anatomical plane. Normally, the femoral artery was located on the upper part of the screen, in the superficial layer, and the femoral vein was located on the lower part of the screen, in the deep layer. The two vessels overlapped slightly (Figure 1). Then, pressing the UPDATE button switched the view to the right screen, the probe rotated anticlockwise by 90° in the same position, and the trainees could quickly identify the femoral vein due to the tissue structure and the depth of the vein as displayed on the left screen. The needle was inserted at a 45° angle using the in-plane technique, and the puncture needle was kept in the center of the long axis of the ultrasound picture. When the puncture needle entered the femoral vein, dark red blood flowed out after suction. The end of the puncture needle was inserted into the tube connected to the infusion device. (The saline injection had been prepared in advance. A needle was initially connected to the infusion device and then replaced with a tube, and the gas was expelled.) When the infusion was observed to have occurred successfully, a guide wire was inserted, and the puncture needle was withdrawn. Ultrasound was used to check that the guide wire was in the femoral vein. Then the skin was stretched, and a deep venous catheter was inserted. Finally, the catheter was locked with heparin water and taped into position.

### 2.3. Observation indices

The number of successful punctures at first attempt, the puncture and catheterization operation time, the number of accidental arterial punctures, and the number of complications such as hematoma formation were recorded. The puncture time was defined as the time from the beginning of skin contact with a sterile probe by the resident to the time of deep venous catheter fixation.

### 2.4. Statistical methods

Data were analyzed using SPSS 20.0 statistical software. Measurement data were expressed as “mean [SD]” and compared between the two groups using the *t*-test for continuous data or the Chi-square test for count data.

## 3. Results

### 3.1. General data

In the test group, the average age was 30 years, 11 were male and 19 female; in the control group, the average age was 29 years, 12 were male and 18 female. The differences in age and gender of residents between the two groups were not statistically significant (*P* > 0.05). The Body Mass Index (BMI) of patients in two groups was 24.2 and 24.5, indicating no statistical difference between the two groups (Table 1). Additionally, none of the patients received antithrombotic therapy before puncture.

**TABLE 1:** General data of resident doctors and patients in two groups.

Item	Control group (*n* = 30)	Observation group (*n* = 30)	Statistics (χ^2^/*t*)	*P*-value
**Gender of residents**				
Male	12 (40%)	11 (36.7%)	0.071	>0.25
Female	18 (60%)	19 (63.3%)		
Age of residents	28.8 (26–32)	30.1 (27–32)	1.297	0.205
BMI of patients	24.5 ± 1.68	24.2 ± 1.64	0.704	0.487

BMI, Body Mass Index.

### 3.2. Results from the questionnaire

There was no significant difference between the two groups in terms of whether they had completed femoral vein puncture independently before, whether they had conducted ultrasound-guided venipuncture before, and their answers to the questions regarding the adjacent positioning of the femoral vein and artery and complications of femoral vein puncture (*P* > 0.05). Therefore, the two groups were comparable (Table 2).

**TABLE 2:** Questionnaire survey results of resident doctors in two groups.

Item	Control group (*n* = 30)	Test group (*n* = 30)	Statistics (χ^2^/*t*)	*P*-value
Have you independently completed femoral vein puncture			0.218	0.640
Yes	2	3		
No	28	27		
Have you conducted ultrasound-guided venipuncture			0	0
Yes	0	0		
No	30	30		
Adjacent relationship between the internal femoral vein and the internal femoral artery			0.480	0.488
Right	24	26		
Wrong	6	4		
Puncture complication			0.218	0.640
Yes	28	27		
No	2	3	−	−

### 3.3. Comparison of puncture results

The success rate at the first puncture attempt in the test group was significantly higher than that in the control group (*P* < 0.05). In the control group, two doctors accidentally punctured the artery, while in the test group, no arterial punctures occurred. The puncture time for the test group was longer than for the control group (Table 3). In addition, some patients in the two groups had a small amount of bleeding at the puncture point, including two cases in the observation group and seven cases in the control group.

**TABLE 3:** Comparison of puncture results of resident doctors between two groups.

Item	Control group (*n* = 30)	Test group (*n* = 30)	Statistics (χ^2^/*t*)	*P*-value
Times of puncture attempt to success			12.36	<0.005
One attempt to success	11	24		
Two attempts to success	9	5		
Three attempts to success	9	1		
Mispuncture of femoral artery			2.069	0.150
Yes	2	0		
No	28	30		
Total puncture time (minute)	8.17 ± 0.972	9.82 ± 1.109	6.565	<0.001

## 4. Discussion

Central venipuncture is a basic clinical skill that all clinicians should master, and it is also an important part of the standardized clinical training for resident doctors. Femoral vein puncture is a relatively simple and safe deep vein puncture operation, so it is suitable for beginners (4), and it is often applied in the treatment of critically ill patients or for emergency hemodialysis. Therefore, it is a basic technique that resident medical doctors need to master. Many previous studies have revealed that the incidence of complications in adult central venous catheterization is 2–15%. Ultrasound-guided central venous catheterization provides a safe and effective positioning method for clinical practice, which can greatly reduce the occurrence of complications and shorten the time for catheterization (5–7). Ultrasound-guided venipuncture can significantly improve the safety and efficiency of venipuncture, even in children where catheterization is difficult (8, 9). Guidelines issued by the British National Society for Clinical Assessment recommend the use of ultrasound-guided selective central venipuncture and catheterization in both children and adults. It also recommends that doctors should consider this method when emergency puncture catheterization is required (10–12).

How to help resident doctors quickly master the ultrasound-guided puncture technique is an important topic to be discussed in clinical teaching. At present, the common guidance on ultrasound methods includes using the short-axis, long-axis, or oblique-axis planes (13). The short-axis method allows the operator to clearly see the tissues and structures around the vein, and the artery and vein can be located more accurately. This is especially important in the process of venipuncture and catheterization, as the relative position of the artery and vein can be clearly seen. However, the disadvantage is that it is difficult to track the insertion of the puncture needle on the ultrasound image, and the position of the puncture needle can only be estimated based on the compression and deformation of the tissue in the ultrasound image. It is also necessary to adjust the orientation of the probe from time to time to see the needle. Therefore, the operator needs to master the operation of the ultrasonic probe and the puncture needle very proficiently and continuously adjust the probe so that the femoral vein and the puncture needle tip can be clearly seen on the image. The long-axis method allows the operator to observe the needle insertion traveling along the long axis of the ultrasound and the long axis of the blood vessel, and during the entire puncture process, it can be clearly seen that the puncture needle passes through the skin, and subcutaneous tissue and enters the vein. However, using this method alone cannot quickly determine whether the blood vessel in the ultrasound plane is the artery or the vein and cannot display the anatomy around the blood vessels. Therefore, using the long-axis method alone requires higher skill levels from the operator. The oblique axis plane method requires short-axis rotation for repositioning, and there is a risk of accidentally puncturing the artery in the plane. This makes it difficult for resident doctors who are learning this technique.

The Department of Critical Medicine of the Air Force Characteristic Medical Center has summarized experience from clinical teaching and innovatively decided to apply the short–long axis switching double-screen contrast method for ultrasound-guided puncture in the standardized training and teaching of resident doctors. This can enable resident doctors to quickly master the skills of femoral vein puncture, improve the success rate of puncture, increase the resident doctors’ confidence in clinical operations, and increase their interest in learning, so it is worth promoting.

The short–long-axis switching double-screen contrast method for puncture teaching adopts ultrasound double-screen display, where the short-axis plane of the left screen can help to quickly determine the artery and vein by enabling comparison of their appearance and pulsation. At the same position, the probe is rotated 90°, and the target blood vessel is quickly determined by the depth of its position as shown on the left screen, and then the long axis is used for puncture guidance. Compared with teaching the traditional ultrasound-guided short–long-axis femoral vein puncture, teaching resident doctors the modified femoral venipuncture technique has improved the total success rate of femoral vein puncture and the success rate of first puncture attempts, reduced the rate of accidental arterial injury, and improved the resident doctors’ self-confidence. Having self-confidence increases the likelihood of success when performing venipuncture.

In summary, using the short–long-axis switching double-screen contrast method to guide deep vein puncture reduces the risk of invasive operations in clinical medical teaching, and better protects patient safety providing an effective teaching method for the standardized training of internal resident medical doctors and achieving good outcomes.

## Data availability statement

The original contributions presented in this study are included in the article/supplementary material, further inquiries can be directed to the corresponding author.

## Ethics statement

The studies involving human participants were reviewed and approved by the Committee of Air Force Characteristic Medical Center, Air Force Medical University. The patients/participants provided their written informed consent to participate in this study.

## Author contributions

YZ and JL: conception and design of the research. YZ and YS: acquisition of data. YZ and WB: analysis and interpretation of the data. WG, YS, and TL: statistical analysis. YM: obtaining financing and critical revision of the manuscript for intellectual content. YZ: writing of the manuscript. All authors contributed to the article and approved the submitted version.
